# BounTI (boundary‐preserving threshold iteration): A user‐friendly tool for automatic hard tissue segmentation

**DOI:** 10.1111/joa.14063

**Published:** 2024-05-17

**Authors:** Marius Didziokas, Erwin Pauws, Lars Kölby, Roman H. Khonsari, Mehran Moazen

**Affiliations:** ^1^ Department of Mechanical Engineering University College London London UK; ^2^ Developmental Biology and Cancer Research and Teaching Department, UCL Great Ormond Street Institute of Child Health University College London London UK; ^3^ Department of Plastic Surgery, Sahlgrenska University Hospital University of Gothenburg Gothenburg Sweden; ^4^ Department of Maxillofacial Surgery and Plastic Surgery, Necker—Enfants Malades Hospital, Assistance Publique—Hôpitaux de Paris; Faculty of Medicine Université Paris Cité Paris France

**Keywords:** 3D reconstruction, bone, computed tomography, craniofacial system, craniosynostosis, image processing, skull

## Abstract

X‐ray Computed Tomography (CT) images are widely used in various fields of natural, physical, and biological sciences. 3D reconstruction of the images involves segmentation of the structures of interest. Manual segmentation has been widely used in the field of biological sciences for complex structures composed of several sub‐parts and can be a time‐consuming process. Many tools have been developed to automate the segmentation process, all with various limitations and advantages, however, multipart segmentation remains a largely manual process. The aim of this study was to develop an open‐access and user‐friendly tool for the automatic segmentation of calcified tissues, specifically focusing on craniofacial bones. Here we describe BounTI, a novel segmentation algorithm which preserves boundaries between separate segments through iterative thresholding. This study outlines the working principles behind this algorithm, investigates the effect of several input parameters on its outcome, and then tests its versatility on CT images of the craniofacial system from different species (e.g. a snake, a lizard, an amphibian, a mouse and a human skull) with various scan qualities. The case studies demonstrate that this algorithm can be effectively used to segment the craniofacial system of a range of species automatically. High‐resolution microCT images resulted in more accurate boundary‐preserved segmentation, nonetheless significantly lower‐quality clinical images could still be segmented using the proposed algorithm. Methods for manual intervention are included in this tool when the scan quality is insufficient to achieve the desired segmentation results. While the focus here was on the craniofacial system, BounTI can be used to automatically segment any hard tissue. The tool presented here is available as an Avizo/Amira add‐on, a stand‐alone Windows executable, and a Python library. We believe this accessible and user‐friendly segmentation tool can benefit the wider anatomical community.

## Graphical abstract

BounTI can be used to segment various skeletal features. In this work, we introduced the novel tool and focused on the segmentation of the craniofacial system in various species. While some limitations of the tool were highlighted in this work in general it was capable of capturing most of the anatomically relevant structures in one of the most complex parts of the skeletal system.

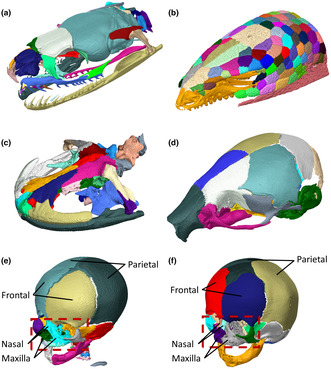

## 1 INTRODUCTION

3D image analysis is extensively used in various fields of natural, physical and biological sciences (e.g. Cunningham et al., 2014; Garvey, 2002; Metzger et al., 2015; Rawson et al., 2020; Razmkhah, 2020). A key part of such analysis involves segmentation, based on computed tomography (CT) or magnetic resonance imaging (MRI). Segmentation is the partition of an image into multiple parts, usually based on voxel characteristics. A number of techniques for segmentation can be used, such as manual selection, grey value thresholds or artificial intelligence (AI) algorithms (e.g. Lenchik et al., 2019). Manual segmentation specifically is an extremely time‐consuming process. Choosing a grey value threshold allows the operators to select the section of the image, with voxel intensities higher than the threshold. This relatively time‐efficient operation can produce acceptable results for bone segmentation as bone tends to have a higher voxel intensity than the surrounding soft tissues (e.g. Ranefall & Wählby, 2016; Singh et al., 2022).

While direct threshold can be an extremely helpful tool to obtain bone segmentation from volume data, it is highly dependent on the image quality (resolution, contrast, sharpness and absence of artefacts). When a single object is considered such as a single bone, a simple threshold may be sufficient to separate the bone voxels from the surrounding soft tissue. However, anatomical structures are usually complex and consist of several hard tissue sub‐parts connected via soft tissue joints. Disarticulating such connected sections can often be laborious and require manual de/selection of the connected voxels and is further complicated in disease models and patient scans with morphological abnormalities (Mansoor et al., 2015).

To facilitate automatic segmentation a variety of tools have been developed, aiming to streamline the process and minimise the required manual user input. These tools included automated threshold selection algorithms (e.g. Otsu, 1979) and local threshold algorithms (e.g. Sauvola & Pietikäinen, 2000) that can help augment the traditional threshold approach. Newer AI algorithms can generate an anatomically accurate segmentation result even when the information in the images is not sufficient to obtain such segmentation through conventional algorithms (e.g. Cui et al., 2022). However, the former is still highly reliant on image quality while the latter often requires extensive training datasets (Belvedere et al., 2022; Engelkes, 2021; Ileșan et al., 2023; Irimia et al., 2019; Rad et al., 2013; Singh et al., 2022; van Eijnatten et al., 2018). Additionally, most of these tools tend to be technically advanced and can be difficult to use for operators.

The aim of this study was to develop and introduce an easy‐to‐use, technically simple, and entirely explicit tool for the automatic segmentation of hard tissues: BounTI (Boundary‐preserving Threshold Iteration). Here we describe the novel algorithm behind BounTI, test its sensitivity to some of its key input parameters and showcase its application to automatically segment and separate different components of the skeleton in different species with a specific focus on the craniofacial region. Nonetheless, the tool developed can be used for the automatic segmentation of other articulated skeletal regions.

## 2 MATERIALS AND METHODS

**FIGURE 1:**
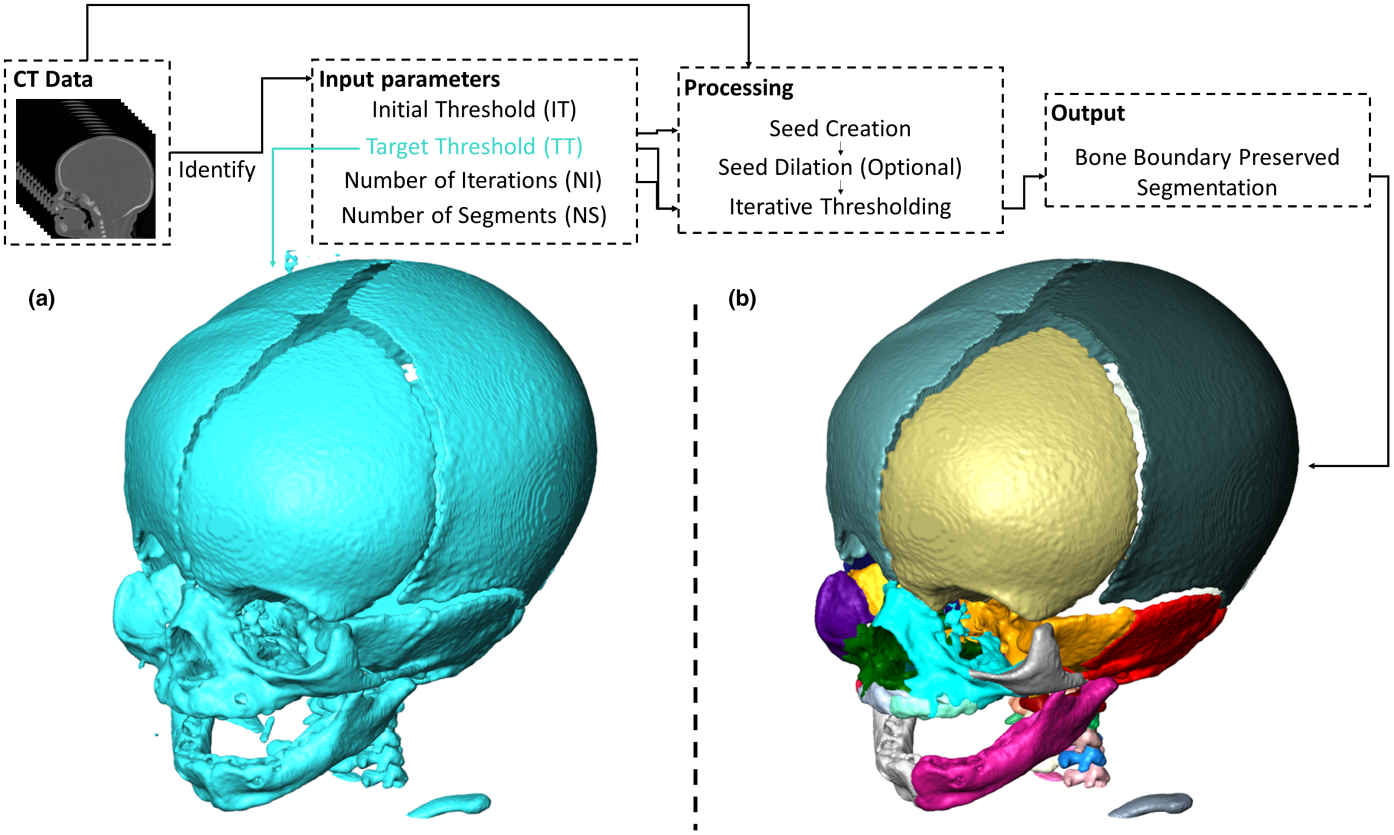
An overview of the technique developed, and threshold‐based versus BounTI segmentation. (a) segmentation using the target threshold (27,000) and (b) segmentation using BounTI (IT – 35,000, TT – 27,000, NI – 150 and NS – 40).

BounTI iterates through thresholds and slowly builds segments onto the seed generated from the initial threshold (Figure 1). It is specifically designed to be accessible to as wide an audience as possible and thus is available as an add‐on for the Avizo/Amira suite, a Python package and a stand‐alone Windows application. The tool and full user guides, including tutorials and further segmentation examples and visualisations, are available in Supplement 1. All of the visualisations in this work were produced through Avizo 2022.1 (Thermo Fisher Scientific, MA, USA). Neither the stand‐alone executable nor the Python library have built‐in visualisation modules, thus, visualising the segmentation results and introducing further manual segmentation for non‐Avizo/‐Amira users will require third‐party software.

### 2.1 BounTI algorithm

#### 2.1.1 Input parameters

The algorithm takes volume data and four numerical variables defined by the operator as input. These include:

- The initial threshold (IT) – a high grey scale value above which the segmentation results in the desired separation of anatomical elements,
- The target threshold (TT) – a low grey scale value above which the segmentation results in the desired bone definition,
- The number of iterations (NI) – a number of threshold steps from the initial threshold to the target threshold that will be done and
- The number of segments (NS) – a number of largest separate segments that will be retained for processing from the initial threshold.

The two parameters that are the most challenging to select are the initial threshold and the number of iterations. Thus, several sensitivity tests were performed on the choice of these parameters on a sample dataset to investigate the effect of the aforementioned parameters.

#### 2.1.2 Processing

BounTI first creates the seed from the initial threshold. The seed here is a segmentation of fully disarticulated components obtained using the initial threshold where the largest separate segments are extracted. The number of these segments in the seed is equal to the number of segments parameter. Each segment in the seed can be optionally expanded by one voxel to include surrounding voxels (dilated), this can produce cleaner results. This option was not employed in these investigations as it can also lead to less predictable results. The threshold step (TS) is the amount by which the threshold will be changed between iterations and is obtained using the following Equation (1.

(1) $$\mathrm{TS}=\frac{\mathrm{IT}-\mathrm{TT}}{\mathrm{NI}}.$$

The volume data is thresholded using a current iteration threshold (CIT) given by the following Equation (2.

(2) CIT=IT−TS×CI.

Here CI is the current iteration. The surrounding connected voxels are added to all of the seed segments individually and the current iteration is increased by one starting from zero. This is repeated until the current iteration is equal to the number of iterations and the last threshold is done at a threshold equal to the target threshold. This results in segmentation that can simultaneously keep the separation achieved by using a high threshold (that alone results in extremely poor definition) and the segment definition that can be obtained by using a low threshold (that alone results in erroneously connected segments).

### 2.2 BounTI tool

The algorithm devised in this work has been implemented into an easy‐to‐use tool available in three distinct forms (Avizo/Amira add‐on, Python library, Stand‐alone Windows executable). While in‐depth user guides are available in Supplement 1. This section briefly introduces the input and output data types, common considerations and highlights the differences between the three implementations of the algorithm.

**FIGURE 2:**
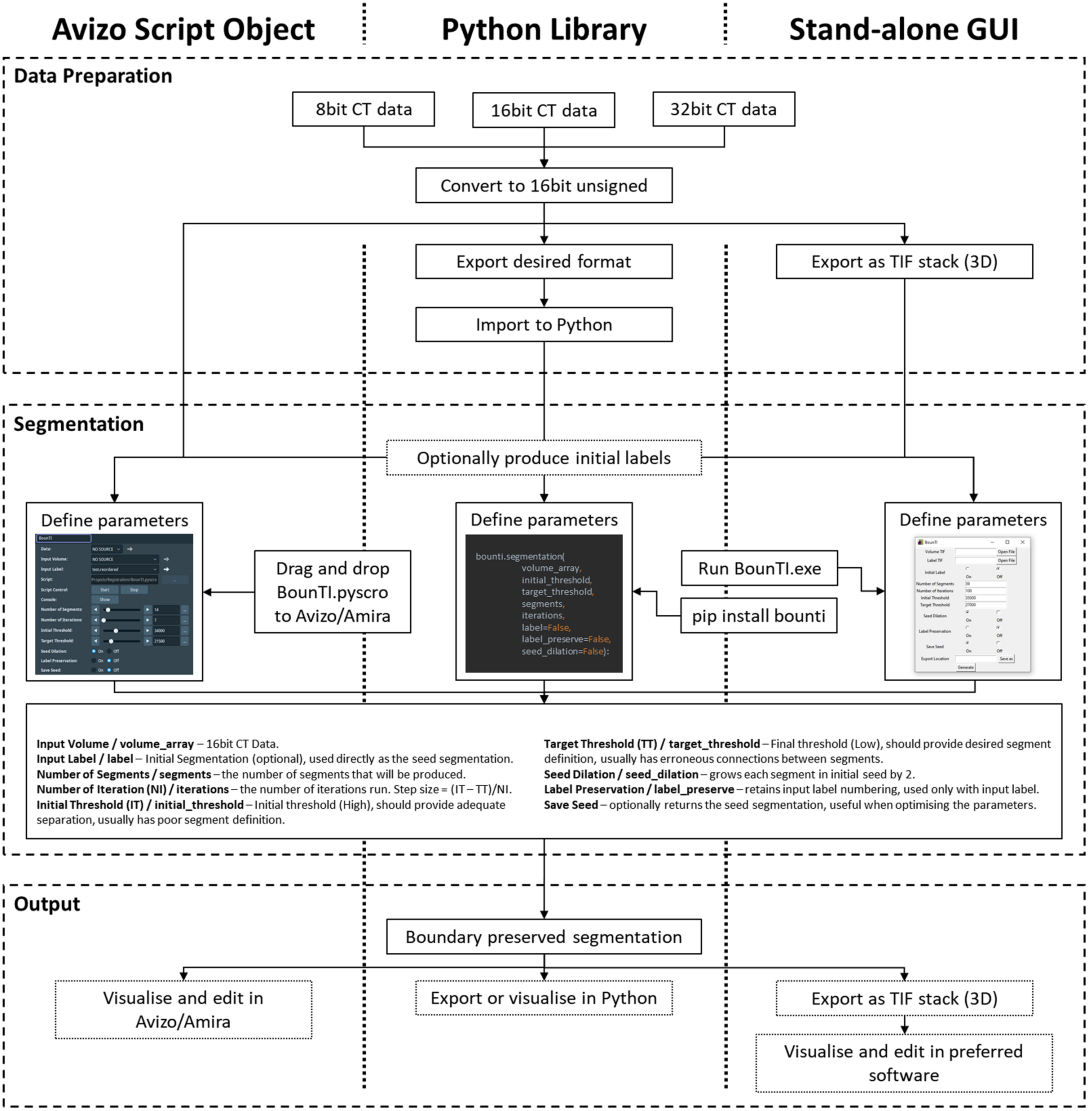
An overview of the three implementations of the algorithm. Highlights the data input and output types, and the different data import and export steps for the Avizo/Amira add‐on, Python library and the Stand‐alone executable.

The workflows for the three implementations are shown in Figure 2. Data preparation can be done in any preferred image processing software. Input CT data should be converted to 16‐bit unsigned format. When converting from 32‐bit data the input range often requires adjustments to minimise bone grey value information loss. As 8‐bit data is rarely used in research settings the tool is not designed to take in such data. Thus, 8‐bit data should be converted to 16‐bit unsigned data with the output range set from 0 to 255. Additionally, it is often advisable to resample the data to at least be under 2Gb, this ensures timely segmentation, which can be particularly important when fine‐tuning the input parameters.

If the Avizo/Amira add‐on is used the data preparation can be conducted in Avizo/Amira, if it is conducted in other software packages any data type that Avizo/Amira accepts can be used. The Python package uses 3D NumPy array (dtype ‐ np.uint16) as input, thus the users can use any external library to convert the data from any data type to the array. A built‐in method is available to import 3D tiff stacks. The stand‐alone executable requires the 3D tiff stack as input data.

While the tool can significantly reduce the segmentation time, manual postprocessing is often a recommended final step. The Avizo/Amira add‐on produces segmentation data that the users will be familiar with and will be able to manipulate as any other segmentation. Similar to the input data type, the Python function will output the same data type, again external libraries can be used to visualise or export the segmentation or a built‐in method is available to export the segmentation as a 3D tiff stack. The stand‐alone executable will export the output as a 3D tiff stack.

The manual in Supplement 1 includes a detailed step‐by‐step guide on how to achieve the best results from specific CT data, highlights some possible use cases and shows how the tool can be used in conjunction with manual segmentation. While all the steps are laid out in Avizo the same steps should apply to any other image segmentation tool.

### 2.3 Volume data

The volume data used in this work were CT images of the craniofacial region. This was chosen as it is a complex system consisting of several bones joined together by cranial and facial sutures and synchondroses (e.g. Richtsmeier and Flaherty, 2013). Hence, it is ideal to test the functionality of BounTI.

First, a preoperative craniosynostosis patient skull (female right‐sided coronal suture fusion, 92 days old) was used to highlight the sensitivity of the BounTI to the choice of its input parameters. Second, to establish the versatility of BounTI, five additional scans were used. These included: a snake (*Lamprophis olivaceus*, adult; Natural History Museum, UK), a lizard (*Pseudopus apodus*, adult; Evans Lab., UK), an amphibian (*Andrias japonicus*, adult; Kanagawa Prefectural Museum of Natural History, JP), a mouse (Mus musculus, 7 days old; Pauws Lab, UK) and a healthy anonymised human skull (female, 111 days old; Necker‐Enfants Malades University Hospital, France).

### 2.4 Sensitivity tests

Three sensitivity tests were performed on the preoperative craniosynostosis patient scan to investigate the effects of input parameters on the segmentation results:

*Initial threshold sensitivity:* the initial threshold for this scan was varied from 30,000 to 40,000 in increments of 1,000. These were set to capture the full range of the bone grey values from the scan. At threshold values lower than 30,000, the soft tissues and noise were included while at values higher than 40,000 little to no bone would be selected. The number of segments was set to 28 as this was deemed sufficient to capture the 23 cranial bones (Dixon et al., 1997) as well as the mandible and vertebrae present in the scan. The number of iterations was adjusted to retain a threshold step of 50, which was done to remove the influence of step size on the outcome. Thus, the segmentations with higher initial thresholds would require more iterations (see Equation (1), if the number of iterations was left unchanged it would result in different step sizes between iterations. Here the target threshold of 27,000 was used for this scan given that it resulted in the desired bone definition.

*Number of iterations sensitivity:* the number of iterations defines how many times the algorithm should be repeated between the initial and target thresholds. Tested values were 10, 25, 50, 100, 150 and 200 resulting in threshold steps of 800, 320, 160, 80, 53 and 40 respectively. These were chosen to capture the full range of the CT contrast resolution, that is, the step size of 40 was finer than the actual spread of the grey values, meaning that further increasing the number of iterations would have had no effect on the segmentation results. An initial threshold of 35,000 (obtained from the initial threshold sensitivity test) was used with the target threshold set to 27,000. The number of segments was set to 28.

*File size sensitivity:* Resampling was used to obtain volumes ranging from 7 MB to 1.2 GB in file size, by increasing or decreasing the voxel size of the original scan. (Lanczos resampling in Avizo) Execution time was taken as the wall time (the amount of time that the program takes from start to finish) on a Windows machine with 128 GB RAM, Intel Xeon W‐2265 CPU @ 3.50 GHz. Algorithm parameters were as follows: 35,000, 27,000, 10 and 28 for the initial threshold, target threshold, number of iterations and number of segments respectively (Table 1).

**TABLE 1:** BounTI parameters used for the different sensitivity tests carried out.

Test	Initial threshold	Target threshold	Number of iterations	Number of segments	File size, MB
Initial threshold sensitivity	30,000–40,000	27,000	60–260	28	181
Number of iterations sensitivity	35,000	27,000	10–200	28	181
File size sensitivity	35,000	27,000	10	28	7–1200

### 2.5 Versatility analysis

**FIGURE 3:**
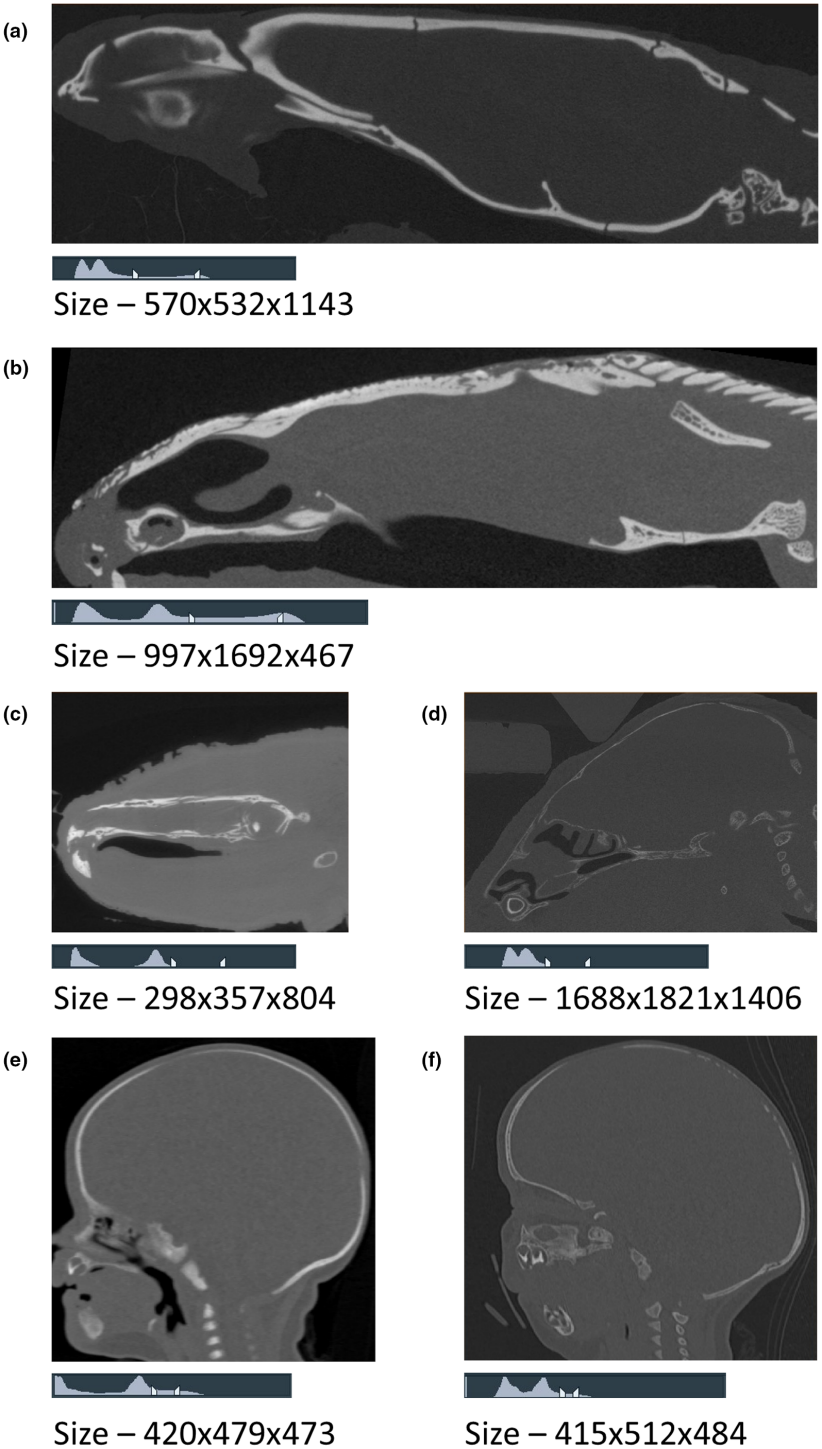
Sagittal slices and scan volume size in voxels for the segmented cases. The histogram range is from 0 (left) to 65,535 (right) and pointers initial (higher) and target (lower) thresholds used for example segmentations. (a) Snake – Lamprophis olivaceus adult, (b) Lizard – Pseudopus apodus adult, (c) Amphibian – Andrias japonicus adult, (d) Mouse – Mus Musculus 7 days old, (e) Human – Female right‐side coronal suture fusion 92 days old, (f) Human – Female 111 days old. Note visualisations in this figure were generated in Avizo image processing software.

BounTI segmentation was performed on all the specimens described in the volume data section. The scans were obtained from different CT machines with different resolutions and qualities. Figure 3 presents a sagittal slice of the aforementioned scans as well as the grey value distribution (left – 0, right – 65,535, range markers show the initial [higher – left] and target [lower – right] threshold used for each case study in the versatility analysis) and volume dimensions in voxels. The initial threshold was selected as the value at which the bones were sufficiently separated when using a direct threshold and the target threshold was the value at which the desired bone definition was reached. As the CT data was arbitrarily converted from 32‐bit to 16‐bit with the goal of preserving the greyscale value data for the bone, the numeric values are arbitrary and have little physical meaning beyond higher values signifying higher x‐ray attenuation. Specific BounTI and scan parameters used for each specimen were as follows:

*Lamprophis olivaceus* (*Snake*): BounTI – Initial threshold of 40,000, target threshold of 21,000, number of segments set to 40 and number of iterations set to 100; Scan – *13 μm × 13 μm × 13 μm*, *90 kV*, *111 μA*, *0.354s exposure*.

*Pseudopus apodus* (*Lizard*) (Marghoub et al., 2023): BounTI – Initial threshold of 48,000, target threshold of 28,000, number of segments set to 200 and number of iterations set to 200; Scan – *23 μm × 23 μm × 23 μm*, not known to us.

*Andrias japonicus* (*Amphibian*) (Matsumoto et al., 2024): BounTI – Initial threshold of 47,000, target threshold of 32,000, number of segments set to 40 and number of iterations set to 100; Scan – *100 μm × 100 μm × 100 μm*, not known to us.

*Mus musculus* (*Mouse*) (Didziokas et al., 2024): BounTI – Initial threshold of 34,000, target threshold of 21,000, number of segments set to 30 and number of iterations set to 100; Scan – *9.6 μm × 9.6 μm × 9.6 μm*, *90 kV*, *90 μA*, *1 s exposure*.

*Homo Sapiens* (*Human*), *female right‐sided coronal suture fusion*, *92 days old* (Mellgren et al., 2024): BounTI – Initial threshold of 35,000, target threshold of 27,000, number of segments set to 28 and number of iterations set to 100; Scan – *293 μm × 293 μm × 300 μm*, not known to us.

*Homo Sapiens* (*Human*), *female*, *111 days old* (Liang et al., 2023): BounTI – Initial threshold of 29,000, target threshold of 22,000, number of segments set to 28 and number of iterations set to 100; Scan – 304 μm *×* 304 μm *×* 296 μm, not known to us.

## 3 RESULTS

### 3.1 Initial threshold sensitivity

**FIGURE 4:**
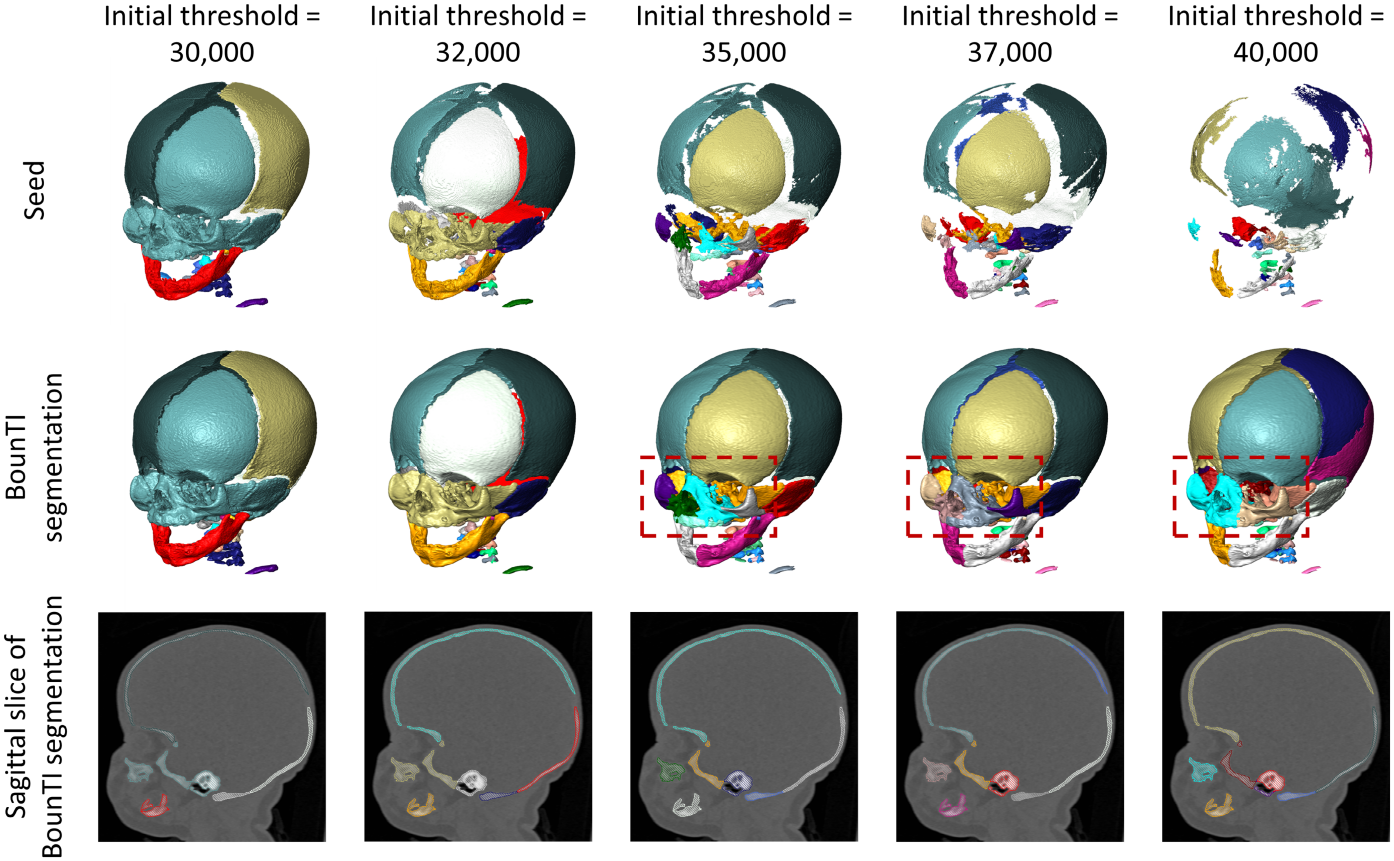
Effects of initial threshold value on the generated seed segments and the final segmentation. (Number of segments set to 28 and the number of iterations adjusted for each threshold to retain a step size of 50). Boxes outline the facial region (nasal, maxilla and zygomatic bones).

The analysis showed that a lower initial threshold resulted in erroneously connected segments because the segments were connected in the seed (Figure 4). Conversely, an overly high initial threshold led to erroneous separation as seen on the parietal bone in the 40,000 threshold case. Additionally, in the latter case, some segments were also erroneously connected due to the seed lacking a segment to build onto (see parietal bone in Figure 4). For brevity five out of the 11 cases tested are shown in Figure 4. The full set is available in Supplement 2. Note, the colours of the segments in all figures were based on the volume of the segment in the seed. This resulted in different colours of the same bone in some of the BounTI segmentation. While this can be easily changed manually or algorithmically to coincide between segmentations in this work it was chosen to present the results exactly as generated.

### 3.2 Number of iterations sensitivity

**FIGURE 5:**
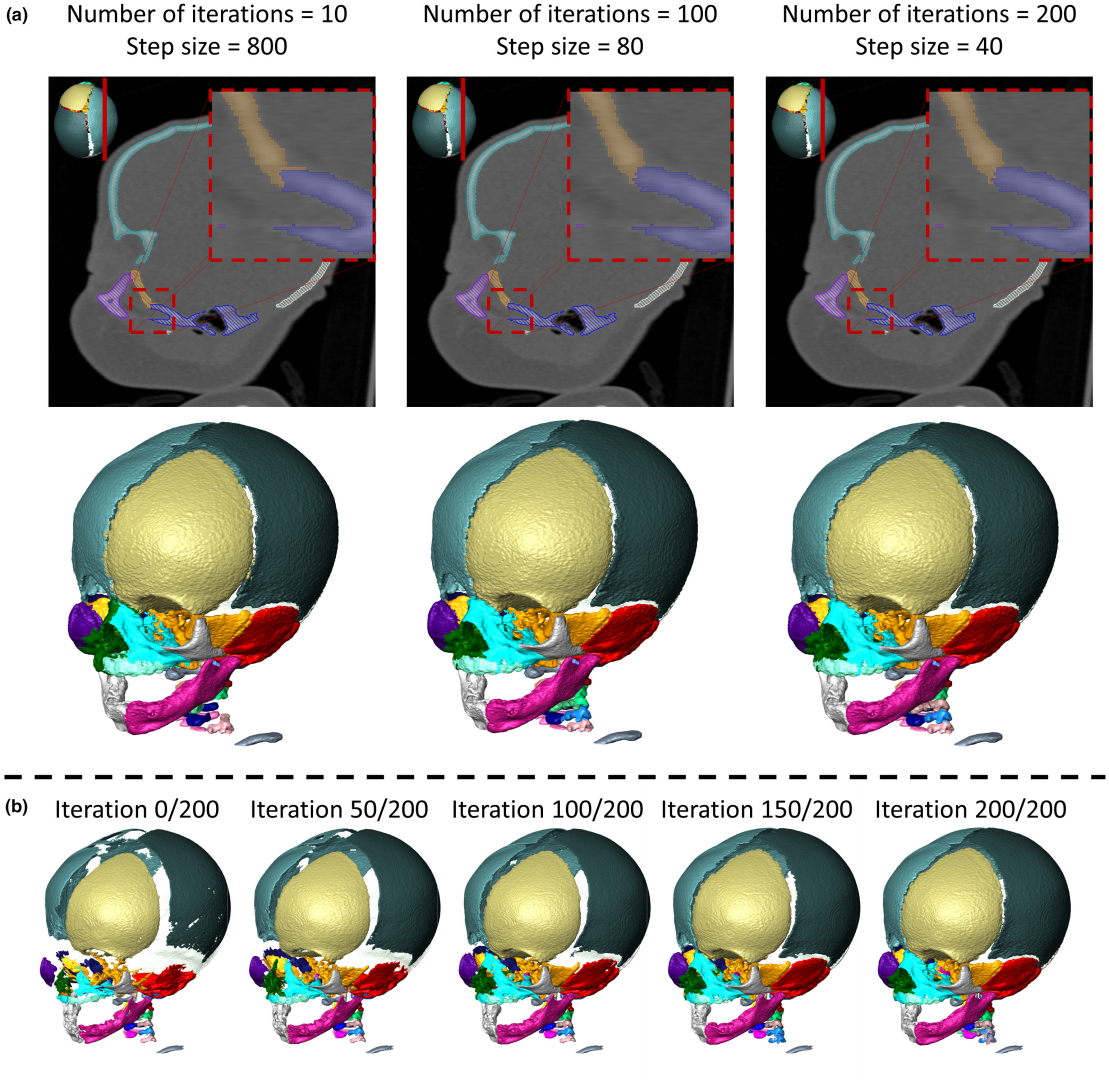
(a) Effect of the number of iterations on segment boundary quality. Boxes outline the interface of two segments and (b) change in the segmentation with iterations completed with an initial threshold of 35,000, target threshold of 27,000, number of segments set to 28, and number of iterations set to 200, resulting in a step size of 40.

The effects of the number of iterations is shown in Figure 5a. The main effect was the definition of the boundary between two adjacent regions, with the higher number of iterations resulting in a cleaner, more anatomically accurate boundary. However, the differences in the boundary between the 100 and 200 iterations cases were hard to identify, suggesting a limit on the boundary‐improving effect of the number of iterations used. For brevity, three out of the six cases tested are shown in Figure 5a. The full set is available in Supplement 3.

Additionally, Figure 5b highlights the working principles of the algorithm showing the outcome of the segmentation through the iterative process, at different iterations where the number of iterations was set at 200 that is, at 0 (0%), 50 (25%), 100 (50%), 150 (75%) and 200 (100%). Animated visualisation for the iterations for both the craniosynostosis case and the normal skull scans is available in Supplement 4.

### 3.3 File size sensitivity

**FIGURE 6:**
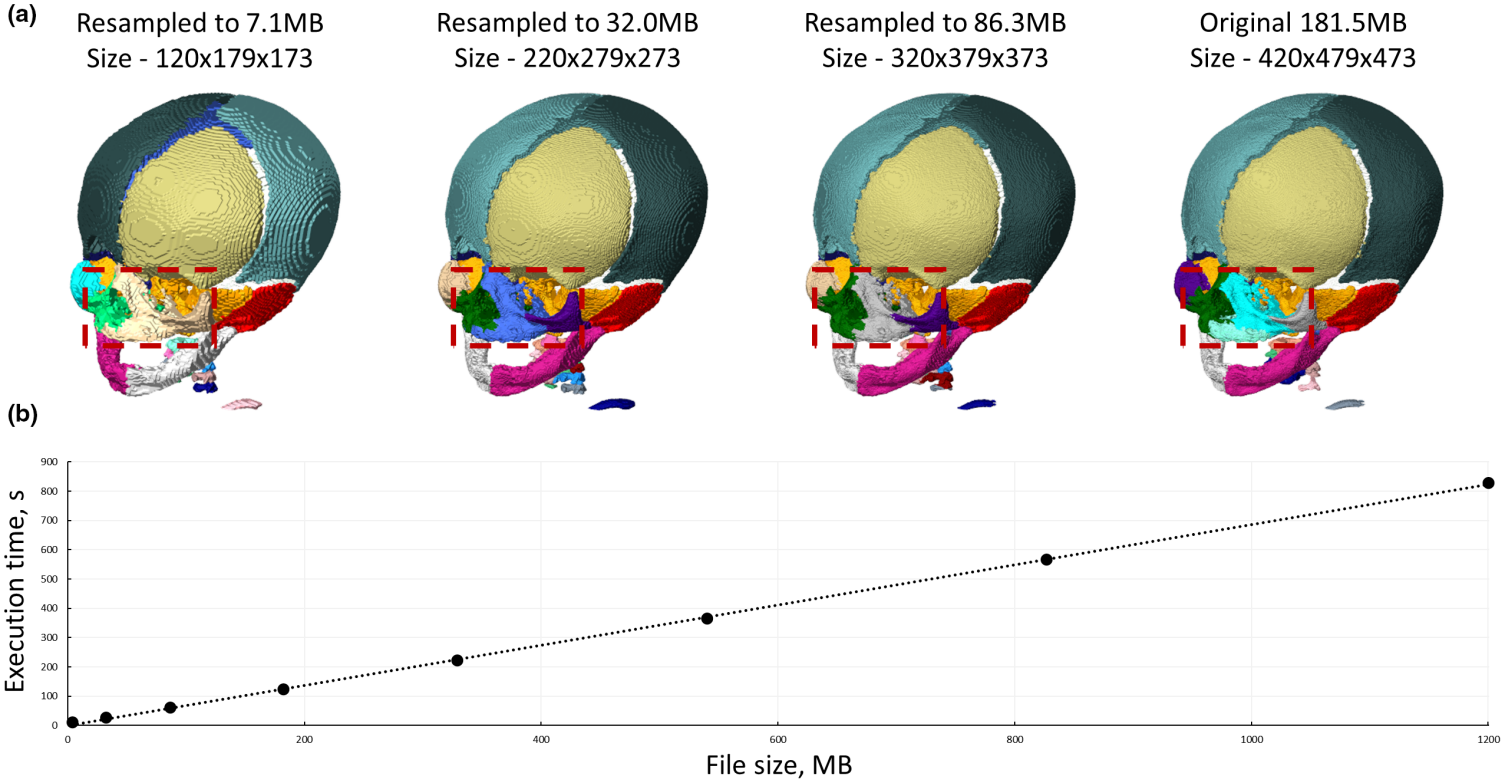
(a) Comparison of segmentation results for downsampled CT data and (b) Execution time in seconds against CT data set size in megabytes. All tests were segmented with an initial threshold of 35,000, target threshold of 27,000, number of segments at 28 and number of iterations set to 10, resulting in a step size of 800.

Some loss of segmentation quality occurred when the volume data were down‐sampled, namely the facial bones (nasal and maxilla) Figure 5a. Overall bone definition was also affected and can be observed as more apparent voxels in the down‐sampled cases. Additionally, the execution time was directly proportional to the file size when the data was down‐sampled (see Figure 6). While the specific relation shown here will not be true for all data sets and BounTI settings, the direct relation between computation time and file size is always present, meaning that as file size increases execution time will also increase.

### 3.4 Versatility analysis

**FIGURE 7:**
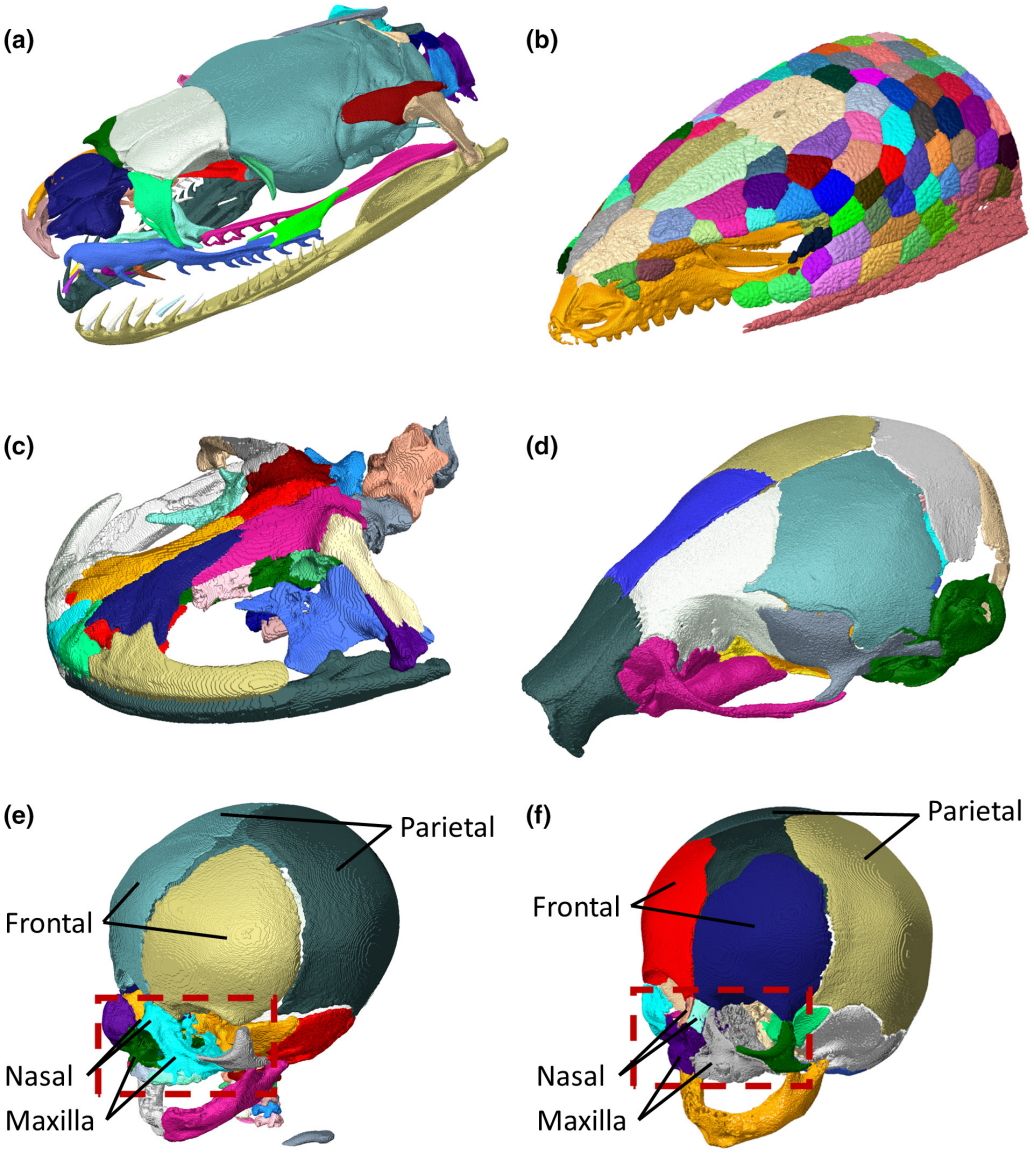
Segmentation results for different CT scans (a) Snake ‐ Lamprophis olivaceus adult, (b) Lizard ‐ Pseudopus apodus adult (c) Amphibian – Andrias japonicus adult, (d) Mouse – Mus musculus 7 days old, (e) Human – Female right‐sided coronal suture fusion 92 days old, (f) Human – Female 111 days old. Boxes outline the facial region (nasal, maxilla and zygomatic bones) in the human segmentations. Bones of interested are highlighted.

The algorithm successfully segmented various bony parts of the craniofacial skeleton in all presented cases (Figure 7). Nonetheless, the results were still dependent on both the specimen type and scan quality and not all the bones were separated even if, anatomically, the bony segments were distinct. This can be observed to some extent in all cases considered here and may still require some manual segmentation. However, the results also highlight the versatility and capabilities of the approach with the majority of the bones segmented and, in the lizard case, including all the individual osteoderms (Figure 7b).

The comparison between the segmentation of the craniosynostosis infant and the normal infant skulls (i.e. Figure 7e vs 7f) highlights two key aspects of the algorithm. Firstly, the fused frontal and parietal bones were not segmented in the former specimen. Additionally, the latter specimen showed better and more anatomical segmentation as seen in the nasal and maxillary bones (see highlight boxes in Figure 7e,f). The unclear boundaries in the craniosynostosis skull segmentation are known as overflow and are a direct result of the scan quality (contrast, resolution and sharpness).

## 4 DISCUSSION

We have here presented a novel automated segmentation tool and highlighted its capabilities. BounTI was successfully developed and used to segment craniofacial bones of different species. The ‘right’ choice of input parameters for this algorithm will depend on a range of parameters including the quality of volume data, specifically resolution and greyscale value distribution (contrast). The user manual available in Supplement 1 can be a useful resource to identify the input parameters. Nonetheless, the sensitivity tests included in this study can act as a troubleshooting guide for users when selecting these parameters for their specific data sets.

The sensitivity tests demonstrated that the segmentation results are primarily affected by the choice of initial threshold. The initial threshold first identifies the seed, that is, produces the initial segmentation from which each segment is then grown. Thus, it had a profound effect on the separation (i.e. segmentation) obtained from the algorithm. A low threshold led to insufficient separation in the initial seed and therefore erroneous connections between the segments. On the other hand, an overly high threshold led to more disconnected segments in the initial seed and thus erroneous disconnections between the segments in the final segmentation. Additionally, the high threshold led to missing segments as the threshold was too high to obtain segments in the initial seed in certain regions. This is observed in Figure 4 (see highlight box) for the 40,000 threshold case where nasal and maxilla bones lack the separation seen in the 35,000 and 38,000‐ threshold cases.

A direct segmentation from a high threshold could be used to obtain the initial threshold parameter. If the segmentation shows connected segments where they are expected to be anatomically separate the initial threshold should be higher, and if some segments are missing the initial threshold should be lower. Additionally, the tool developed here allows the direct extraction of the created and optionally dilated seed, which can then be investigated and used to adjust the initial threshold accordingly.

Some volume data may not have sufficient resolution, contrast, or sharpness to obtain a well‐separated seed with enough definition to build the segments in the iteration process. In such cases, the initial seed can be put in manually, instead of using the generated seed from the initial threshold. If this approach is employed then the initial seed can either be preserved that is, the segment assignments retained, or disregarded by selecting the largest disconnected segments. For the latter method, all the desired segments should already be disconnected in the seed, while using the former will retain the segment separation even when the segments are in contact (connected). This option was not investigated in this work, more details on how to perform seed manipulation are available in Supplement 1.

The number of iterations sensitivity test did not display a large difference between the values considered due to the volume data used. The spread of the grey value range (across the bone voxels see Figure 3e histogram) in the initial data was relatively small (Figure 5b). This significantly limits the effect of increasing the number of iterations. However, when comparing the 10 and 100 iterations cases a clear difference in the quality of the boundaries between the segments can be seen with the higher iteration producing a more anatomical boundary definition. This was not further improved by increasing the number of iterations to 200 iterations (see highlight boxes in Figure 5b). This analysis suggests that the number of iterations that should be selected is affected by the spread of grey values across the bone. The spread is dependent on the resolution, contrast and level of noise in the scan among other factors. An overly high number of iterations may not necessarily improve the quality of the final segmentation results, but it will impact the computational time. For example, in a lower file size volume (e.g. clinical CT) this may not be of concern. However, for larger volumes, it can considerably impact the computational time.

The file size sensitivity test showed that a larger file required more computational time (Figure 6b). Volume data file size can be reduced by three main methods. First, the grey values can be converted to use fewer bits. In the case of this algorithm, unsigned 16‐bit data is required as input, thus 32‐bit, signed 16‐bit, and 8‐bit data should be converted to unsigned 16‐bit data. For the 8‐bit case, the output should be from 0 to 255 when converting. Secondly, the volume of interest can be cropped to include only the desired parts for segmentation. Lastly, the data may be down‐sampled, however, as shown in Figure 6a this can impact the quality of the final segmentation. Down‐sampling can lead to the largest decrease in computational time and should be considered when using extremely large microCT volumes such as those presented in Figure 3d.

Lastly, the versatility is highlighted in Figure 7 as the approach can be used for a variety of species as well as a range of initial volume qualities as seen in Figure 3. This suggests that BounTI can be an invaluable tool for a range of disciplines from anatomical research to surgical planning as segmentation has been a historically tedious and time‐consuming manual process. As a manual target threshold is used in the algorithm for the final threshold, it can never yield any selection different to one segmented with the same threshold directly. Consequently, in the worst‐case scenario, BounTI will have separated meaningless segments, but the overall definition will be exactly as it would have been using direct thresholding. However, in all cases shown here the majority of the segments separated are anatomically accurate and useful.

Nonetheless, BounTI has limitations. The comparison between craniosynostosis and normal human skull scans shows that the algorithm cannot separate physically fused bones. More crucially the higher quality of the normal human scan highlights the importance of scan quality as the segmentation was more anatomically accurate for the normal skull. Namely the more accurate separation of the nasal and maxillary bones in the normal skull compared to the craniosynostosis skull highlighted in Figure 7e,f. A conceptually similar approach attempted to address the overflow issue that has led to these scan quality‐based separation differences in BounTI (Huang et al., 2011). However, this method requires significantly more computational steps and the overflow issue only occurs for low‐resolution and low‐contrast images. Direct threshold iteration was chosen for BounTI to retain the relative simplicity of the algorithm and to maximise the information obtained from the grey values of the volume data directly, but this resulted in lower‐quality segmentation for some clinical CT scans that tend to yield lower resolution/lower contrast images compared to higher‐quality images such as those produced with microCT. The required scan quality largely depended on the specimen investigated as specimens with smaller gaps between bones may require higher resolution or specimens with less difference in the x‐ray attenuation between the bones and tissues connecting the bones may require longer scans to improve contrast.

The focus of this work was on the segmentation of the craniofacial system however, the tool can be used to segment other skeletal regions as is. Segmentation of iodine‐stained soft tissues may be possible using the tool however, the slightly higher grey values observed surrounding the soft tissue boundaries in these scans contradict the core principle of the algorithm that the grey value is lower at the boundary than other parts of the segment. Similar issues are present in fossil scans as well as MRI scans. While it may be possible to segment these scans either with or without additional processing steps using BounTI this has not been tested.

Pilot validation has been included in Supplement 5. This includes a comparison of two extremely different manual segmentation cases, a normal human infant skull to later be used for FEA and a minimally segmented Yucatan 1‐month‐old mini pig where the goal of the segmentation was to separate the segments manually by removing the material in the sutures connecting bones. These validation examples are in no way comprehensive and BounTI users are highly advised to carry out case‐specific sensitivity tests, especially when the segmentation is used directly for analysis as opposed to further manual corrections.

A commonly used score to evaluate segmentation accuracy is the Dice Similarity Coefficient. It captures the similarity between two arrays. In this case, Dice scores were calculated for each segment and the average of these scores presented as the overall similarity between the manual and BounTI segmentation. The average Dice scores for the human scan were 56% and 72% with the missing components in the BounTI segmentation included and not included in the average respectively. For the pig segmentation no components were missing in the BounTI segmentation and the average accuracy was 99%. As FE models require increased suture thickness to be computationally viable the majority of the difference between manual and BounTI segmentation is present across the sutures in the human segmentations. (Supplement 5a) The sutures have been significantly idealised in the manual segmentation giving rise to the majority discrepancy in dice scores between the human and pig segmentation accuracy (Liang et al., 2024). Additionally, some of the segments in the manual human segmentation were not separated in the BounTI segmentation due to insufficient grey value separation of these small facial bones further contributing to the differences in accuracy. This investigation highlights the variability of BounTI segmentation accuracy due to segmentation goals and data quality.

Even with the limitations laid out in this work, BounTI was able to successfully segment the vast majority of the anatomically separate bones in all of the investigated specimens in a matter of minutes compared to days, weeks or in the lizard case months of work required to manually separate the segments. The tool is available in three distinct forms – Avizo/Amira addon (Script Object), Python library and a stand‐alone executable. As the method is shown to be versatile it is crucial to make it as accessible as possible to researchers and clinicians of all backgrounds (see also Davies et al., 2017). With the segmentations produced by BounTI, it is sometimes possible to automatically infer the soft tissue joints connecting the craniofacial bones (see example in Supplement 1). Further investigations into the techniques described in this work may lead to the automation of not only the bone segmentation but also the segmentation of sutures and synchondroses.

## AUTHOR CONTRIBUTIONS

Marius Didziokas: Data collection, Conceptualisation, Methodology, Writing – original draft, Data processing, Coding, Validation. Erwin Pauws: Data collection, Writing – review and editing, Supervision. Lars Kölby: Data collection, Writing – review and editing. Roman H. Khonsari: Data collection, Writing – review and editing. Mehran Moazen: Methodology, Writing – original draft, Supervision.

### OPEN RESEARCH BADGES

This article has earned an Open Data badge for making publicly available the digitally‐shareable data necessary to reproduce the reported results. The data is available at https://github.com/Didziokas/BounTI.

## Supporting information


**Supplementary File 1:** https://github.com/Didziokas/BounTI (Includes tutorials, packages and further visualisation)
Supplement 1: Packages, tutorials and further visualisation (https://github.com/Didziokas/BounTI)
Supplement 2: Effects of the initial threshold value (from 30,000 to 40,000) on the generated seed segments and the final segmentation (number of segments set to 28 and the number of iterations adjusted for each For Peer Review Only threshold to retain a step size of 50).
Supplement 3: Effect of the number of iterations (10, 25, 50, 100, 150 and 200) on segment boundary quality.
With an initial threshold of 35,000, target threshold of 27,000, number of segments set to 28.
Supplement 4: Threshold iteration process for (a) craniosynostosis skull and (b) normal skull. The current threshold is indicated by the red bar across the histogram.
Supplement 5a: Comparison of BounTI and manual segmentation for a normal human infant skull.
Supplement 5b: Comparison of BounTI and manual segmentation for a 1‐month‐old Yucatan mini‐pig skull.

## Data Availability

Source code of the developed tool compiled add‐ons and additional information are freely available at https://github.com/Didziokas/BounTI.
